# The Editor's Letter-Box

**Published:** 1913-01-18

**Authors:** L. H. Glenton Kerr

**Affiliations:** Secretary. Great Northern Central Hospital, Holloway Road, N.


### The Editor's Letter-Box.

### Hospital Almoners and Insured Patients.

*To the Editor of* The Hospital.

Sir,—I have the pleasure to forward copy of the regulations which have been adopted by this hospital in connection with the treatment of insured persons under the National Health Insurance Act, and which may be of interest to your readers.—Yours faithfully,

L. H. Glenton Kerr, Secretary.

Great Northern Central Hospital,
Holloway Road, N.,
January 11, 1913.

GREAT NORTHERN CENTRAL HOSPITAL.
NATIONAL INSURANCE ACT.

Regulations adopted by the Committee of Management, January 2, 1913.

1. All persons coming to the hospital (except in case of urgent illness) shall be asked by an official of the hospital whether or not they are insured.

2. Every insured person shall be referred to a medical officer to decide whether his or her illness is urgent or not.

3. An insured person (except in case of urgent illness) shall, after first aid has been rendered, be referred to his or her own doctor, and shall not be treated again without a certificate in writing (on the form provided by the hospital) from his or her own doctor that the case is one requiring hospital treatment.

4. Notices to be posted in prominent positions in the hospital stating that: (*a*) On and after January 15, 1913, all persons applying for treatment will be required to state whether they are insured or not. (*b*) Insured persons with small ailments will not be treated at the hospital.

